# Use of High-Resolution Geospatial and Genomic Data to Characterize Recent Tuberculosis Transmission, Botswana

**DOI:** 10.3201/eid2905.220796

**Published:** 2023-05

**Authors:** Chelsea R. Baker, Ivan Barilar, Leonardo S. de Araujo, Anne W. Rimoin, Daniel M. Parker, Rosanna Boyd, James L. Tobias, Patrick K. Moonan, Eleanor S. Click, Alyssa Finlay, John E. Oeltmann, Vladimir N. Minin, Chawangwa Modongo, Nicola M. Zetola, Stefan Niemann, Sanghyuk S. Shin

**Affiliations:** University of California, Irvine, California, USA (C.R. Baker, D.M. Parker, V.N. Minin, S.S. Shin);; Forschungszentrum, Borstel, Germany (I. Barilar, L.S. de Araujo, S. Niemann);; University of California, Los Angeles, California, USA (A.W. Rimoin);; US Centers for Disease Control and Prevention, Gaborone, Botswana (R. Boyd, A. Finlay);; US Centers for Disease Control and Prevention, Atlanta, Georgia, USA (J.L. Tobias, P.K. Moonan, R. Boyd, E.S. Click, A. Finlay, J.E. Oeltmann);; Botswana–UPenn Partnership, Gaborone (C. Modongo, N.M. Zetola);; Victus Global Botswana Organisation, Gaborone (C. Modongo, N.M. Zetola)

**Keywords:** tuberculosis and other mycobacteria, bacteria, respiratory infections, whole-genome sequencing, spatial analysis, geographic heterogeneity, outbreaks, infectious disease control, Botswana

## Abstract

Combining genomic and geospatial data can be useful for understanding *Mycobacterium tuberculosis* transmission in high-burden tuberculosis (TB) settings. We performed whole-genome sequencing on *M. tuberculosis* DNA extracted from sputum cultures from a population-based TB study conducted in Gaborone, Botswana, during 2012–2016. We determined spatial distribution of cases on the basis of shared genotypes among isolates. We considered clusters of isolates with ≤5 single-nucleotide polymorphisms identified by whole-genome sequencing to indicate recent transmission and clusters of ≥10 persons to be outbreaks. We obtained both molecular and geospatial data for 946/1,449 (65%) participants with culture-confirmed TB; 62 persons belonged to 5 outbreaks of 10–19 persons each. We detected geospatial clustering in just 2 of those 5 outbreaks, suggesting heterogeneous spatial patterns. Our findings indicate that targeted interventions applied in smaller geographic areas of high-burden TB identified using integrated genomic and geospatial data might help interrupt TB transmission during outbreaks.

Tuberculosis (TB) remains among the leading causes of death from infectious diseases worldwide, killing 1.5 million persons in 2020 despite being preventable and curable (*1*). High-burden TB countries often contend with limited financial and labor resources and rely on generalized interventions that, although helpful, treat TB as a uniform epidemic (*2*–*4*). However, recent advances in molecular methods have shown that TB epidemics are composed of multiple simultaneous chains of transmission that could serve as distinct targets for intervention (*4*–*7*). Targeted interventions to interrupt transmission might be particularly effective for reducing TB in high-burden settings, where recent infections contribute substantially to disease incidence (*3*–*6*).

Genomic sequencing is a powerful tool for identifying discrete, but closely related, *Mycobacterium tuberculosis* strains, helping to reconstruct likely chains of recent transmission (*3*,*8*,*9*). Genomic and geospatial data can be integrated to investigate whether transmission chains fall within distinct geographic areas (*3*–*6*). For example, spatial clusters of closely related *M. tuberculosis* strains may indicate localized areas of ongoing transmission, which could be targeted for public health interventions, such as active case finding (*3*–*5*,*10*). A growing body of evidence suggests that geographically targeted interventions could be effective and cost-efficient in high-burden, low-resource settings and instrumental in accelerating progress toward eliminating TB (*4*,*11*–*13*). 

In the Kopanyo Study, a population-based study of TB transmission in Botswana during 2012–2016, localized transmission events were characterized by detecting spatial clustering of participants belonging to genotype-specific (genotypic) cluster groups identified by using MIRU-VNTR (mycobacterial interspersed repetitive unit–variable number tandem repeat) genotyping (*5*,*14*). The objectives of our analysis were to build on data from the original study by incorporating higher resolution genomic data from whole-genome sequencing (WGS) and to investigate the geographic distribution of distinct genotypic cluster groups representing potential recent transmission chains. The Kopanyo Study was approved by the US Centers for Disease Control and Prevention institutional review board (approval no. 6291), the Health Research and Development Committee of the Botswana Ministry of Health and Wellness, and institutional review boards of the University of Pennsylvania. We received written informed consent from all participants and mapped residential coordinates in sufficiently low resolution to prevent identification of participants.

## Methods

### Study Design and Setting

We analyzed data collected during August 2012–March 2016 for the Kopanyo Study among persons with TB in Botswana, a country in southern Africa with a high burden of TB and TB/HIV co-infection (*1*,*5*,*14*). Nationwide TB incidence when the study began was 305 cases/100,000 persons (*5*,*14*). This analysis included participants residing in greater Gaborone, including the capital city and its surrounding suburbs. During the 5 years before the study, TB incidence was 440–470 cases/100,000 persons in Gaborone, which had a total population of 354,380 (*5*,*14*). 

Study participants included men and women of all ages with TB disease who were sequentially enrolled by date of diagnosis; those who had already received TB treatment for >14 days, prisoners, and patients who declined to participate were excluded (*5*,*14*). At least 1 sputum sample was collected from each participant for bacterial culture. Clinical and demographic data, including residential address, were collected through in-person interviews and medical record review (*5*,*14*). We obtained residential geocoordinates using global positioning system (GPS) devices during site visits or by geocoding addresses using Google Maps (https://www.google.com/maps), OpenStreetMap (https://www.openstreetmap.org), and ArcGIS (Esri, https://www.esri.com) (*5*,*14*).

### WGS

We conducted WGS on archived DNA samples from the original study with sufficient amounts of DNA (>0.05 ng/μL) for analysis. We initially prepared DNA by crude extraction from liquid culture samples as described elsewhere (*15*). We prepared libraries for sequencing using an Illumina Nextera XT kit (https://www.illumina.com) to obtain 2 × 150 bp fragments for paired-end sequencing using a Illumina NextSeq 500 platform (*16*,*17*). To assemble and analyze sequences, we used MTBseq pipeline (https://github.com/ngs-fzb/MTBseq_source), which incorporates several open-source programs, including Burrows-Wheeler Aligner (https://github.com/lh3/bwa), Samtools (http://www.htslib.org), and Genome Analysis Toolkit version 3 (https://github.com/broadinstitute/gatk/releases), to automate steps involved in sample-specific and comparative analyses (*16*,*17*). We mapped reads to the *M. tuberculosis* H37Rv reference genome (GenBank accession no. NC_000962.3) (*16*). We performed variant calling using default thresholds for coverage and quality (*16*). We identified phylogenetically informative single-nucleotide polymorphisms (SNPs) from existing literature (*16*). We annotated variants associated with antimicrobial resistance on the basis of a built-in list of known mutations (*16*,*17*) and generated summaries to predict resistance for each genotype. As an indicator of recent TB transmission, we used a cluster-detection algorithm to identify closely related strains within each lineage (lineages 1–4) based on a distance threshold of ≤5 pairwise SNPs to establish bacterial genetic relatedness (*8*). The single linkage cluster detection algorithm used to identify genotype-specific groups detects isolates within 5 SNPs from the next closest isolate, so not all members within a given group are necessarily within 5 SNPs of all other members (*16*).

### Spatial Analysis

Our main analysis included participants residing in greater Gaborone who had both WGS data and GPS coordinates available. We excluded 29 participants with evidence of possible mixed-strain infection (*18*), which was detected using a method based on a binomial test procedure described elsewhere (*19*). For our analysis, we focused mainly on participants in 5 outbreak groups, defined as groups of >10 persons infected with genotype-specific TB. To represent the underlying density of TB infection in the population, we included ungrouped participants (those not in an identified genotypic group of any size) as a comparison group.

We conducted a preliminary analysis comparing the geographic distribution of participants with and without WGS data available to rule out geographic sampling bias. We estimated the geographic median center point and standard deviational ellipse (directional distribution at 2 SD) for both sets of participants. The median center is a measure of central tendency that is robust to outliers and minimizes the distance from the central location to all other points being analyzed. The standard deviational ellipse encompasses most observed points along both geographic coordinates (latitude and longitude), providing a representation of geographic range and directional orientation. We then used those same methods to characterize the geographic distribution of participants belonging to each outbreak, as well as ungrouped participants. We used a Monte Carlo test of spatial segregation to measure geographic variation among the different outbreak groups (*20*).

We generated kernel density maps to visualize locations of potential spatial clusters. Estimating kernel density provides an estimate of spatial concentration in terms of points per unit area using a moving window method with a weighting scheme and generating a smoothed map that displays areas of greater density (*21*). We generated maps for each outbreak group and for ungrouped strains using a 1-km buffer window. For visual display, density is shown on a different scale for ungrouped (up to 35 persons/km^2^) and grouped participants (up to 5 persons/km^2^) because of differences in size of datasets.

To estimate spatial clustering among participants in each outbreak group, we used spatial K-function analysis, a method that measures whether points are located closer to one another on average than would be expected in a completely random spatial pattern (*21*). To account for potential clustering caused by underlying population density, we compared relative clustering in grouped and ungrouped participants by estimating the difference in K-functions over a range of distances (0–8,000 m) (*21*,*22*). We generated plots with distances indicated along the x axis and K-function estimates along the y axis and examined the shape and behavior of the observed K-function values for interpretation (*21*). We used 999 random permutations to obtain 95% CIs. We assessed the magnitude of lines above or below 0 on the y axis to compare degree of clustering among groups and lines falling outside the upper or lower confidence intervals to detect statistically significant differences. 

We calculated pairwise SNP and geographic distances of participants by outbreak group to assess whether relationships between geographic and genetic difference varied by group and generated boxplots to display SNP distance summaries. We plotted geographic distance against SNP distance and tested for correlation using Spearman ρ. We investigated possible spatial-temporal trends by measuring the geographic distance between the first participant (based on dates documented during the original study) diagnosed with TB and subsequently diagnosed participants in each outbreak group. We plotted date of diagnosis against geographic distance to visualize possible patterns. In addition, we conducted a sensitivity analysis to assess geographic characteristics of genotypic groups obtained using a distance threshold of ≤2 SNPs. For groups defined in this additional analysis, we estimated median center points, directional distributions, and differences in K-functions. We performed initial mapping and descriptive spatial analysis including median center, directional distribution, and kernel density using ArcGIS version 10.7.1 and performed additional analysis and data visualization in R statistical software version 4.1.2 (The R Project for Statistical computing, https://www.r-project.org). We calculated pairwise geographic distances by using R package fields (https://cran.r-project.org/web/packages/fields/index.html) and pairwise SNP distances by using ape (https://cran.r-project.org/web/packages/ape/index.html) (*23*). We used splancs (https://cran.r-project.org/web/packages/splancs/index.html) and smacpod (https://cran.r-project.org/web/packages/smacpod/index.html) for K-function analysis. Boxplots and scatter plots were displayed using ggplot2 (https://ggplot2.tidyverse.org) and egg (https://cran.r-project.org/package=egg) with the viridis (http://www.iqtree.org/https://sjmgarnier.github.io/viridis) color palette. 

### Phylogenetic Analysis

We generated a maximum-likelihood phylogenetic tree using IQ-TREE version 1.6.12 (http://www.iqtree.org) (*24*) to represent genetic relationships among *M. tuberculosis* strains. We specified a Hasegawa-Kishino-Yano substitution model, which allows for unequal base frequencies and unequal transition rates, and corrected for ascertainment bias (*25*). To construct the phylogenetic tree, we used a midpoint rooting approach and expanded our dataset to include all participants with *M. tuberculosis* strains belonging to lineage 4, after excluding isolates with evidence of possible mixed infection. We highlighted the location within the tree of the main outbreak groups in our analysis and vertically expanded branches from the node representing the estimated most recent common ancestor for each group to enable detailed visualization. We then projected phylogenetic trees onto geographic maps for each of the groups, displaying the location in the tree of each *M. tuberculosis* isolate linked with its corresponding geographic location. We used R packages ggtree (https://github.com/YuLab-SMU/ggtree) (*26*), phytools (https://cran.r-project.org/web/packages/phytools/index.html) (*27*), rgdal (https://cran.r-project.org/package=rgdal), mapdata (https://cran.r-project.org/web/packages/mapdata/index.html), and prettymapr (https://cran.r-project.org/package=prettymapr) to annotate and visualize the tree.

### Epidemiologic Links

We analyzed data on occupation, places of employment, and social gathering places (e.g., markets, places of worship, taverns) to provide additional context for interpreting WGS and geospatial data (*28*). We used common occupational groups and social gathering places shared by >2 participants to identify potential epidemiologic links (*28*). 

## Results 

A total of 1,449 participants with culture-confirmed TB and primary residence in greater Gaborone had valid GPS coordinates, of which 946 (65%) had WGS data available and were thus eligible for this analysis (*5*,*14*). We determined that participants with WGS data were geographically representative of participants overall and that distributions of age, sex, HIV status, and income were similar between participants with and without WGS data (Appendix Table 1). We excluded 29 participants with evidence of possible mixed-strain infections. There were 431 participants that belonged to genotype-specific groups of 2–19 persons, including 62 participants belonging to 5 large groups of >10 persons, which we considered outbreaks. Data from the 62 participants comprising outbreak groups A–E and the 486 in a control group of participants who did not belong to any genotype-specific group, a total of 548 participants, were the focus of our primary analysis (Table 1). 

**Table 1:** Characteristics of participants (N = 548) in study of high-resolution geospatial and genomic data to characterize recent tuberculosis transmission, by outbreak group (≤5 SNP), Gaborone, Botswana, 2012–2016*

Category	Group and lineage	Ungrouped, n = 486

A, n = 19	B, n = 12	C, n = 11	D, n = 10	E, n = 10
4.1.1.3	4.1.1.3	4.1.2.1	4.1.1.3	4.3.4.1
Median age, y (IQR)	29 (24–40)	35 (28–40)	33 (31–42)	31.5 (30–37)	39 (34–42)	35 (28–42)
Gender						
M	11 (58)	9 (75)	9 (75)	3 (30)	10 (100)	254 (52)
F	8 (42)	3 (25)	3 (25)	7 (70)	0	232 (48)
HIV status						
Positive	9 (47)	6 (50)	9 (82)	5 (50)	5 (50)	308 (64)
Negative	10 (53)	6 (50)	2 (18)	5 (50)	4 (40)	162 (33)
NA	0	0	0	0	1 (10)	16 (3)
Income						
Any	15 (79)	5 (42)	8 (73)	4 (40)	8 (80)	360 (74)
None	4 (21)	7 (58)	2 (18)	6 (60)	2 (20)	125 (25)
NA	0	0	1 (9)	0	0	1 (<1)
Isoniazid						
Susceptible	16 (84)	12 (100)	11 (100)	10 (100)	10 (100)	458 (94)
Resistant	3 (16)	0	0	0	0	28 (6)
Rifampin						
Susceptible	16 (84)	12 (100)	11 (100)	9 (90)	8 (80)	456 (94)
Resistant	3 (16)	0	0	1 (10)	2 (20)	30 (6)

*Values are no. (%) except as indicated. NA, not available.

Median age among ungrouped participants was 35 years (IQR 28–42 years); 52% were male, 25% reported no income, and 64% had diagnosed TB/HIV co-infection (Table 1). On the basis of genotypic prediction, we estimated that most had *M. tuberculosis* susceptible to first-line antimicrobial drugs isoniazid (94%) and rifampin (94%). Among participants in the 5 genotypic groups, median age ranged from 29 years in group A to 39 years in group E (Table 1). Participants in group E were exclusively men; D was the only group with more women than men (70%); group C had the most participants with diagnosed TB/HIV coinfection (9/11; 82%). The percentage of participants reporting no income ranged from 18% in group C to 60% in group D. Three participants in group A had multidrug-resistant TB with predicted resistance to both isoniazid and rifampin.

**Figure 1:**
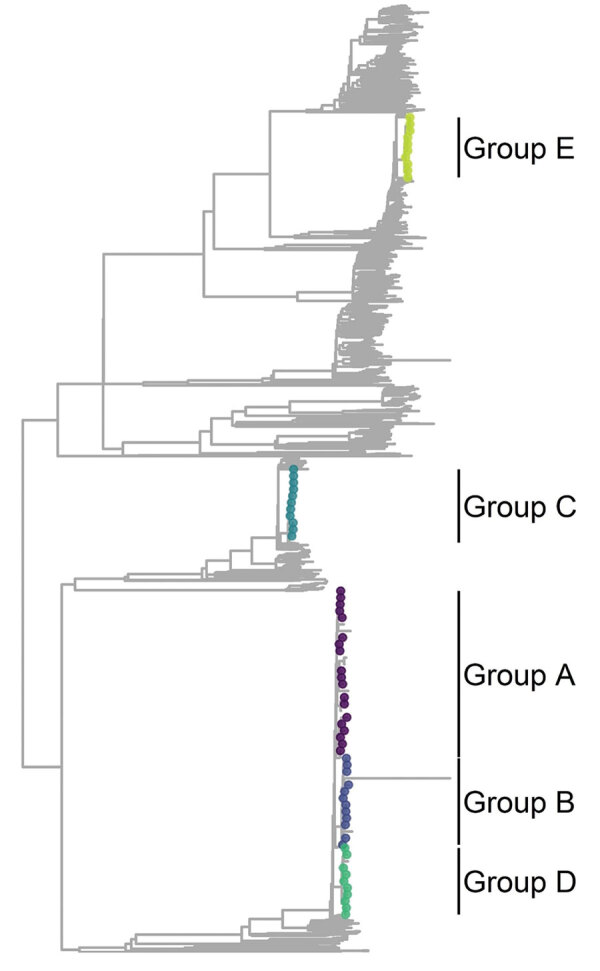
Phylogenetic tree representation for *Mycobacterium tuberculosis* lineage 4 for selected genotypic cluster groups (≤5 single-nucleotide polymorphisms) in study of high-resolution geospatial and genomic data to characterize recent tuberculosis transmission, Gaborone, Botswana, 2012–2016. Colors indicate the location of isolates in each genotypic cluster group. Branches within each of the groups are expanded for visualization.

The maximum-likelihood phylogenetic tree for lineage 4 (Figure 1) shows the genetic location of isolates in each outbreak group, highlighted with different colors corresponding to each group. Groups A, B, and D all belonged to sublineage 4.1.1.3 (Euro-American [X-type]) and were located near each other in the tree; group C belonged to sublineage 4.1.2.1 (Euro-American [Haarlem]) and group E to 4.3.4.1 (American [LAM]) (*29*) and were located at greater genetic distances from the other groups.

**Figure 2:**
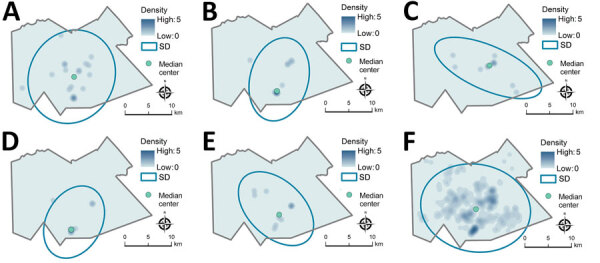
Kernel density map, median center point, and directional distribution for genotypic groups A–E (≤5 single-nucleotide polymorphisms) (panels A–E) and genotypically ungrouped *Mycobacterium tuberculosis* strains (F) in study of high-resolution geospatial and genomic data to characterize recent tuberculosis transmission, Gaborone, Botswana, 2012–2016. The blue ovals encompass the area within the SD ellipse, representing the geographic distance and directional orientation of participant locations within each group. Density is shown on a different scale (up to 35 cases/km^2^) for ungrouped participants than for participants in the genotypic cluster groups (up to 5 cases/km^2^) because of differences in size of the datasets.

**Figure 3:**
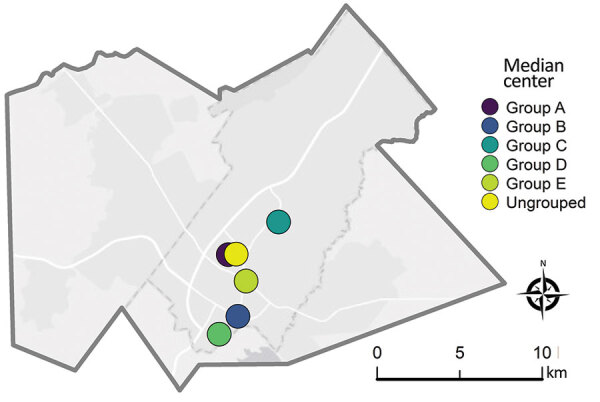
Median center points for *Mycobacterium tuberculosis* genotypic groups A–E (≤5 single-nucleotide polymorphisms) and genotypically ungrouped strains in study of high-resolution geospatial and genomic data to characterize recent tuberculosis transmission, Gaborone, Botswana, 2012–2016. The median center represents a centralized geographic location that is estimated by minimizing the distance to all other participant locations being analyzed.

As displayed in maps showing kernel density estimations, median center points, and directional distributions for each outbreak group and for ungrouped participants (Figure 2), we detected significant spatial segregation among outbreak groups (p = 0.038). There was also spatial segregation among center points for each group (Figure 3) and different directional distributions (Figure 2) among groups. For example, participants in group C were spread over 12 km in an elongated east–west distribution, but groups B and D both had a more compact (<10 km) north–south spread (Table 2; Figure 2). In contrast, residential locations for ungrouped participants were widely spread across the study area (Table 2; Figure 2). The distance between the center points for ungrouped participants and each of the genotypic groups ranged from <0.5 km for group A to ≈5 km for group D (Table 2; Figure 3).

**Table 2:** Spatial summary for each *Mycobacterium tuberculosis* outbreak group (≤5 SNP) in study of high-resolution geospatial and genomic data to characterize recent tuberculosis transmission, by distance rank from reference, Gaborone, Botswana, 2012–2016

Distance rank	Group	Median center distance, m	X span, m	Y span, m	Rotation, degrees
1	Group A	458	9120	10,263	28
2	Group E	1,633	9,597	5,983	134
3	Group C	3,143	12,431	4,768	111
4	Group B	3,788	6,251	9,077	15
5	Group D	4,979	5,946	8,197	29
Referent	Ungrouped	Referent	11,786	9,507	103

**Figure 4:**
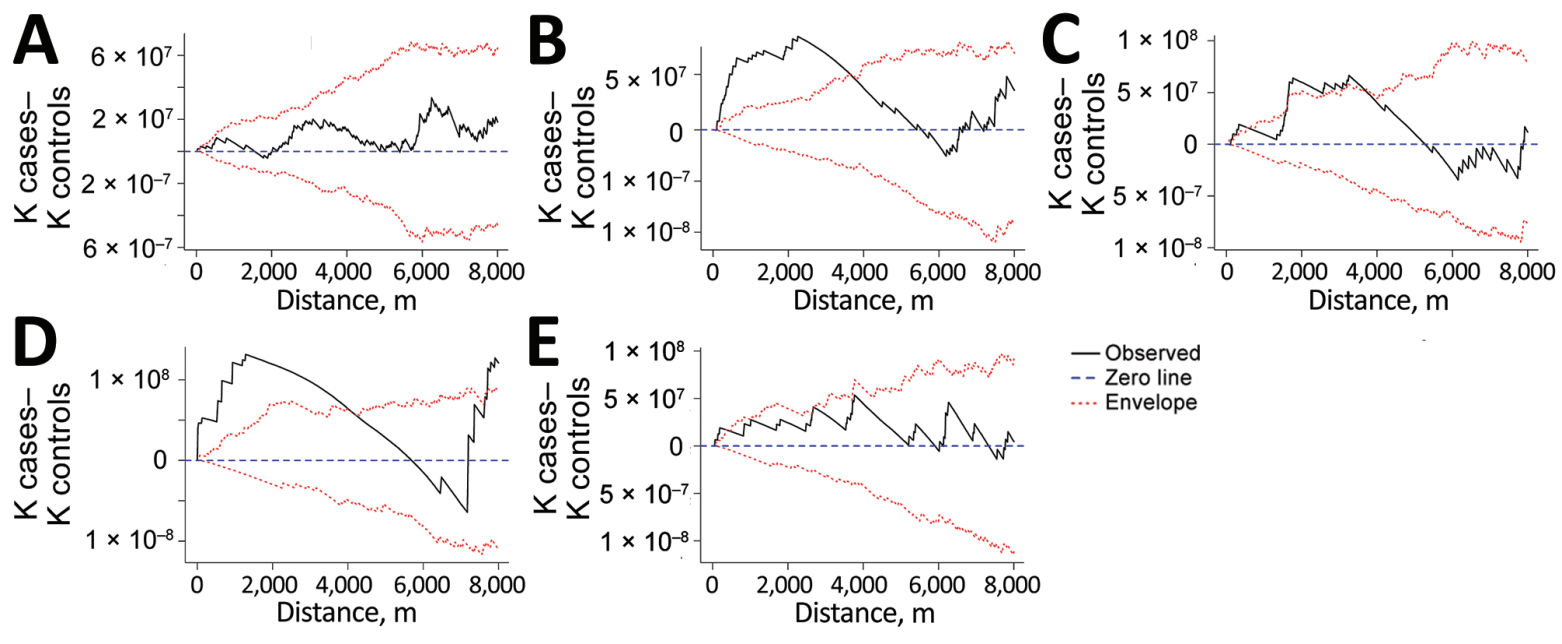
K-function differences for *Mycobacterium tuberculosis* genotypic groups A–E (≤5 single-nucleotide polymorphisms) compared with ungrouped strains in study of high-resolution geospatial and genomic data to characterize recent tuberculosis transmission, Gaborone, Botswana, 2012–2016. Differences in K-functions were used to assess geospatial clustering among participants in each group relative to participants with ungrouped strains. Observations falling above the upper 95% envelope indicate significant spatial clustering.

**Figure 5:**
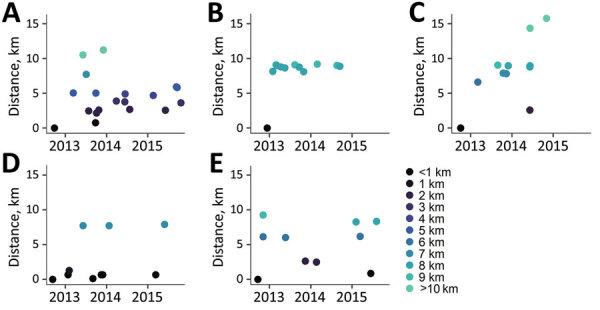
Incident tuberculosis by geographic distance from first study participant by genotypic cluster group (≤5 single-nucleotide polymorphisms) in study of high-resolution geospatial and genomic data to characterize recent tuberculosis transmission, Gaborone, Botswana, 2012–2016. Plots represent each participant by date of tuberculosis diagnosis and by geographic distance (based on participant’s primary residence) from the first participant (shown in each plot at a distance of 0 km) in each genotypic cluster group.

Locations of potential spatial clusters of participants within each group were visually apparent from estimations of kernel density, especially for groups B and D in the south-central part of the map (Figure 2). The presence of spatial clustering in those groups was also supported by results of the K-function analysis (Figure 4). Differences in K-functions indicated participants in groups B and D had significantly greater spatial clustering than participants with ungrouped strains at relatively close distances (up to ≈4 km). Geographic distance between the first and subsequent case diagnoses over time varied by group (Figure 5). Although group C had an overall pattern of increasing distance over time of detection, all subsequently diagnosed cases in group D were located relatively near the first participant; subsequent participants in group B were located at relatively large but equal distances from the first diagnosed case-patient.

**Figure 6:**
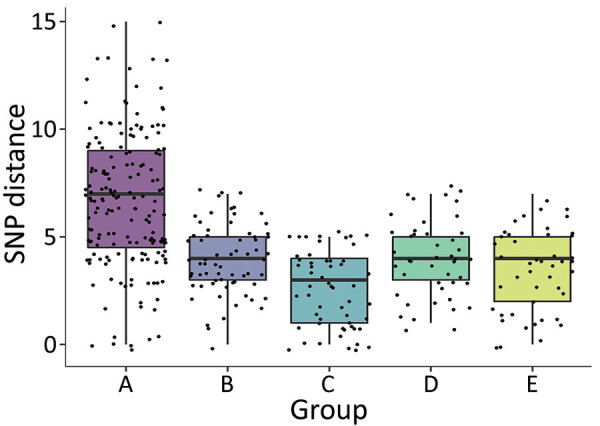
Pairwise SNP distances by ≤5 single-nucleotide polymorphism (SNP) genotypic cluster group in study of high-resolution geospatial and genomic data to characterize recent tuberculosis transmission, Gaborone, Botswana, 2012–2016. Box plots with individual data points superimposed display SNP distance summaries by group. Median within-group SNP distance was <5 SNPs for all groups except group A, which had a median of 7 SNPs. Horizontal lines within boxes indicate medians; box tops and bottoms indicate interquartile ranges; error bars indicate 95% CIs.

**Figure 7:**
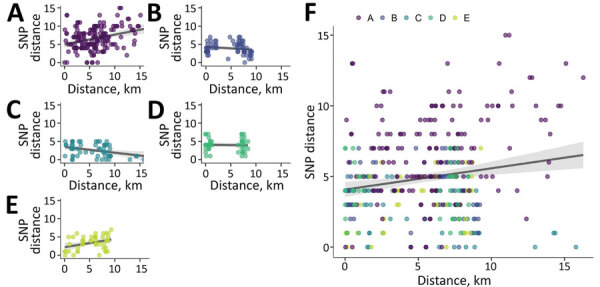
Correlation between pairwise single-nucleotide polymorphisms (SNP) distance and pairwise geographic distance for genotypic cluster groups ≤5 SNP (A–E) and ungrouped cases (F) in study of high-resolution geospatial and genomic data to characterize recent tuberculosis transmission, Gaborone, Botswana, 2012–2016. Points represent measurements for within-group pairs. There was low positive correlation between pairwise geographic and SNP distances overall (Spearman ρ = 0.1; p = 0.06). SNP, single-nucleotide polymorphism.

Median distance within groups was <5 SNPs for all groups except A, which had a median of 7 (Figure 6). Group A also had higher variability in pairwise SNP distances compared with other groups. We observed low positive correlation between geographic and SNP pairwise distances overall (ρ = 0.1; p = 0.06) (Figure 7). However, this correlation varied by group; groups A (ρ = 0.26; p = 0.001) and E (ρ = 0.3; p = 0.045) displayed low to modest positive correlation, whereas group C showed negative correlation (ρ = −0.33; p = 0.015). 

**Figure 8:**
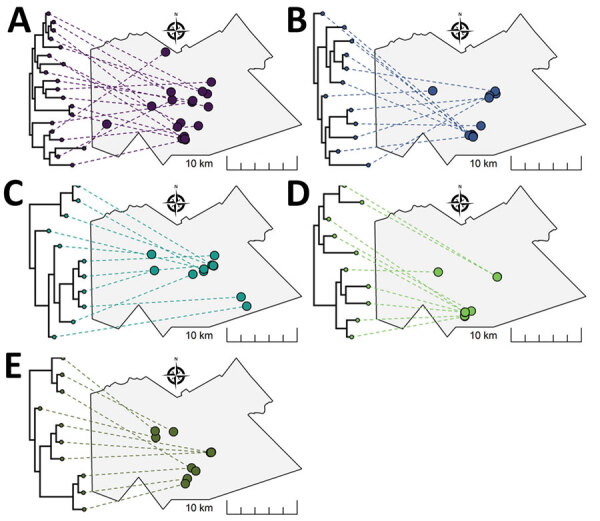
Representation of phylogenetic trees for *Mycobacterium tuberculosis* genotypic cluster groups A–E (≤5 single-nucleotide polymorphisms) projected onto geographic maps in study of high-resolution geospatial and genomic data to characterize recent tuberculosis transmission, Gaborone, Botswana, 2012–2016. The location of each *M. tuberculosis* isolate in the tree is displayed with a link drawn to its corresponding geographic location. Tree tips on the same bifurcating branches represent the most closely related isolates.

Phylogenetic tree displays linked to spatial maps (Figure 8) show heterogenous genotypic and geographic patterns in the different groups. In group E, closely related *M. tuberculosis* isolates were generally located closer in space and separate areas of potential geographic clustering were visible. In group D, most isolates appeared to aggregate in a single geographic cluster, regardless of within-group genetic relatedness. We observed a similar pattern in group B with 2 potential spatial clusters. In groups A and C, closely related isolates were generally dispersed more broadly over the geographic area. We visually identified distinct subclusters of spatially and phylogenetically linked cases in all groups. Potential epidemiologic links were identified in each of the outbreak groups. At least 2 participants in all groups but C had similar occupations (Appendix Table 2). Each group had >1 participant associated with 3–6 social gathering places (Appendix Table 3). In group E, 2 participants with the same occupation also had 2 social gathering sites in common (alcohol-related venues).

Results of the sensitivity analysis indicated genotypic groups defined using a ≤2 SNP threshold also displayed distinct geographic characteristics. Fewer participants overall were identified as belonging to a genotype-specific group using the lower SNP threshold. There were 50 participants total in the largest groups (labeled groups A2–G2, with 6–9 participants each), and 643 ungrouped participants (Appendix Table 2). Similar to our primary outbreak group analysis, those groups displayed significant spatial segregation (p = 0.049), different directional distributions, and spatially varied median center points (Appendix Figure 1). Groups A2, B2, D2, and F2 had significant spatial clustering at shorter distances (0.5–4.0 km).

## Discussion

In our analysis, outbreak groups of patients infected with closely related *M. tuberculosis* strains displayed distinct geospatial characteristics. Less genetic and spatial heterogeneity among participants in 2 of the outbreak groups might indicate localized areas of more recent transmission compared with outbreak groups that were less closely spatially clustered, which might reflect a more advanced stage in the transmission trajectory. Geographic distance between first and subsequent cases varied by group. The first case in group B was located at a relatively large but equal distance from all subsequent cases. Further mapping efforts could incorporate direction as well as distance to subsequent cases to help examine whether the first case may have potentially introduced TB to >1 areas of localized transmission. However, this observation could alternatively be explained by timing of recorded sampling, missed cases, or incomplete spatial data. A location-based approach using the most recently diagnosed instead of the first diagnosed case has been suggested as an alternative, high-yield approach for active case finding (*30*).

Our results support findings from a previous analysis (*5*) that found evidence of localized transmission by detecting spatial clustering of genotypic groups identified using MIRU-VNTR typing. Although overall areas of spatial aggregation were similar, our analysis incorporated higher-resolution genomic sequencing data to detect finer-scale spatial patterns and describe the geographic distribution of distinct genotypic groups. Our results also align with recent studies combining spatial and WGS data to study TB transmission in several other high-burden settings, including China (*31**,**32*), Ghana (*33*), and along the Thailand-Myanmar border (*34*). Observed spatial patterns among related *M. tuberculosis* strains have included local and regional distributions of outbreak groups (*31*,*33*) and lineages (*34*), and associations between residential proximity and genetic similarity (*31*,*32*). In contrast, a study in China found that the majority of genotypic groups included participants from separate geographic districts (*28*). However, that study differed from ours because it specifically analyzed multidrug-resistant TB, and 70% of participants had migrated from other provinces (*28*).

Phylogenetic trees and geographic maps are often presented as complementary but separate displays of data. We generated phylogenetic trees linked to spatial maps that produced a high-resolution display for each genotypic cluster that could guide public health activities. For example, potential subgroups of closely related strains within outbreak groups could be linked with corresponding geographic locations to help identify high-risk areas for targeted interventions, including active case finding for early diagnosis and treatment, contact investigations, and TB-prevention therapies. 

Multiple strata of data are missing from our analysis that might have affected results on detection and geospatial characterization of outbreak groups. Although the original study had relatively high enrollment (4,331/5,515 persons diagnosed during the study period), not every person with TB was captured, including those diagnosed but not enrolled and an unknown number of undetected cases. We excluded participants not culture-confirmed (n = 2,169), which reduced the sample size but helped ensure persons misdiagnosed with TB were not included in the analysis. WGS results were available for culture-confirmed participants with samples containing sufficient DNA (n = 1,426). We further excluded participants for whom we had no geographic coordinates and those with possible mixed-strain infections (*18*,*35*). More complete data could have led to detecting larger or additional outbreak groups or alternate geospatial patterns. However, we believe that the data available are representative of the largest genotypic clusters in the study area and reflect real geographic patterns. We also did not have detailed social contact data. Although we did analyze occupational and social gathering data to identify potential epidemiologic links, additional WGS and epidemiologic data incorporating spatial and social network analysis might have helped us better reconstruct potential transmission chains (*36*).

In conclusion, integrating genomic and geospatial data presents a promising approach for studying TB transmission in high-burden settings. We used this approach to identify heterogeneity among multiple *M. tuberculosis* transmission chains. We identified geographically clustered strains of *M. tuberculosis* representing localized areas of recent transmission. Although barriers remain, substantial progress has been made toward increasing capacity for genomic technologies in low- and middle-income countries (*37*,*38*). Integrated genomic/geospatial combined approaches used in near real time could help TB-prevention programs identify emerging outbreaks and plan and mobilize interventions to interrupt ongoing transmission (*37*,*38*).

Appendix Additional information on study combining geospatial and genomic identity to track tuberculosis transmission in Gaborone, Botswana.
