# Supplementary material for: Use of High-Resolution Geospatial and Genomic Data to Characterize Recent Tuberculosis Transmission, Botswana
Source: Emerg Infect Dis. 2023 May;29(5):977–87. doi: 10.3201/eid2905.220796 (PMC10124643; doi:10.3201/eid2905.220796)
Supplement: Appendix — Additional information on study combining geospatial and genomic identity to track tuberculosis transmission in Gaborone, Botswana. [file 22-0796-Techapp-s1.pdf]

Article DOI: https://doi.org/10.3201/eid2905.220796

EID cannot ensure accessibility for supplementary materials supplied by authors. Readers who have difficulty accessing supplementary content should contact the authors for assistance.

Use of High-Resolution Geospatial and Genomic Data to Characterize Recent Tuberculosis Transmission, Botswana

Appendix

Appendix Table 1. Characteristics of participants with primary residence in Gaborone with and without WGS data available.

Characteristic	No WGS; n = 504	WGS; n = 945	Overall; n = 1,449
Median age, y	32.0	33.0	33.0
Sex			
F	232 (46.0%)	421 (44.6%)	653 (45.1%)
M	272 (54.0%)	524 (55.4%)	796 (54.9%)
HIV			
Negative	195 (38.7%)	370 (39.2%)	565 (39.0%)
Positive	295 (58.5%)	543 (57.5%)	838 (57.8%)
Not available	14 (2.8%)	32 (3.4%)	46 (3.2%)
Income			
Any	348 (69.0%)	660 (69.8%)	1008 (69.6%)
None	154 (30.6%)	278 (29.4%)	432 (29.8%)
Not available	2 (0.4%)	7 (0.7%)	9 (0.6%)

Appendix Table 2. Occupation category by genotypic groups (≤5 SNP), Gaborone, Botswana, 2012–2016.

Category	A; n = 19	B; n = 12	C; n = 11	D; n = 10	E; n = 10	Overall; n = 62
Agriculture/environmental	0	0	0	0	2 (20.0%)	2 (3.2%)
Cleaner/housekeeper	0	1 (8.3%)	1 (9.1%)	0	0	2 (3.2%)
Construction/general labor	3 (15.8%)	1 (8.3%)	1 (9.1%)	2 (20.0%)	1 (10.0%)	8 (12.9%)
Driver	2 (10.5%)	0	0	0	0	2 (3.2%)
Mechanic/machine operator/technician	1 (5.3%)	2 (16.7%)	1 (9.1%)	0	0	4 (6.5%)
Office worker/manager	0	0	0	0	1 (10.0%)	1 (1.6%)
Other	1 (5.3%)	0	1 (9.1%)	0	1 (10.0%)	3 (4.8%)
Personal services	0	0	1 (9.1%)	0	0	1 (1.6%)
Police/security	1 (5.3%)	0	1 (9.1%)	1 (10.0%)	2 (20.0%)	5 (8.1%)
Restaurant worker	3 (15.8%)	0	0	0	1 (10.0%)	4 (6.5%)
Retail/vendor/manufacturing	0	1 (8.3%)	1 (9.1%)	1 (10.0%)	0	3 (4.8%)
Self-employed	3 (15.8%)	0	0	0	0	3 (4.8%)
Student	1 (5.3%)	0	0	2 (20.0%)	0	3 (4.8%)
Unemployed	4 (21.1%)	7 (58.3%)	4 (36.4%)	4 (40.0%)	2 (20.0%)	21 (33.9%)

Appendix Table 3. Shared social gathering locations (≥2 participants) by genotypic group (≤5 SNP), Gaborone, Botswana, 2012–2016.

	Number of shared locations by type			
Group	Markets/shopping centers	Transport hubs	Alcohol-related venues	Total
A	3	0	0	3
B	4	2	0	6
C	5	0	1	6
D	3	1	0	4
E	1	1	2	4

Appendix Table 4. Genotypic cluster groups (≤2 SNP), Gaborone, Botswana, 2012–2016.

Group (≤2 SNP)	No. of participants	Sublineage
Group A2	9	4.1.1.3
Group B2	8	4.1.1.3
Group C2	8	4.1.2.1
Group D2	7	4.3.4.1
Group E2	6	4.1.2
Group F2	6	4.2.4.2.1
Group G2	6	3.1.1

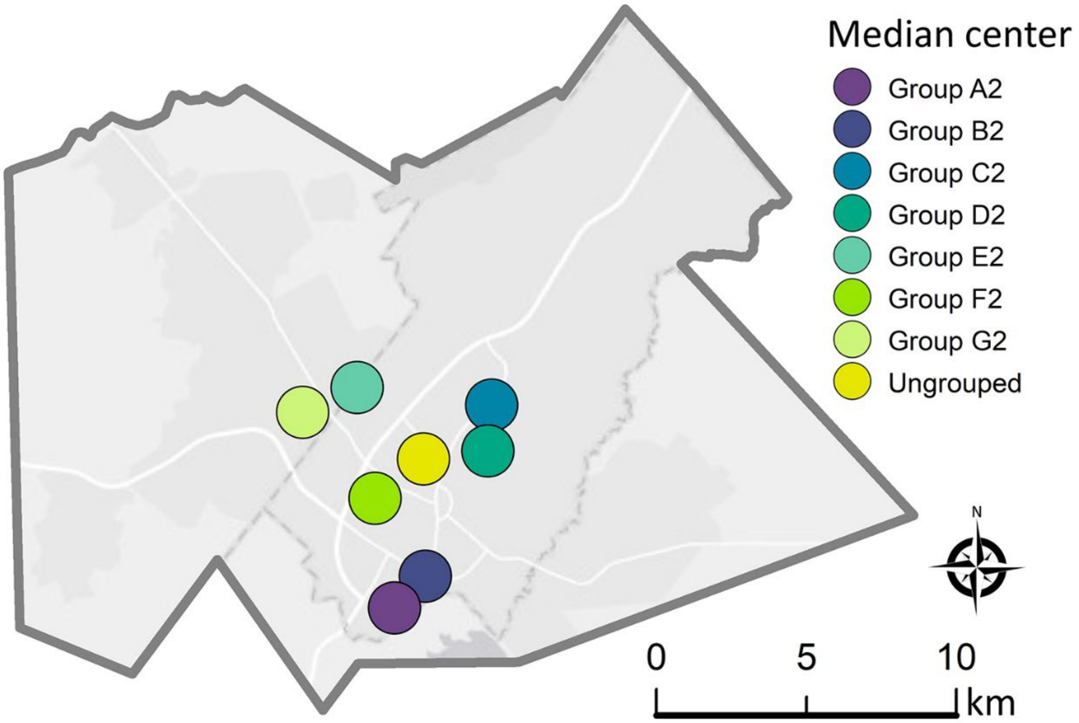

Appendix Figure. Median center points for genotypically grouped (≤2 SNP; groups A2–G2) and ungrouped *M. tuberculosis* participants in study of genetic and geographic characteristics of tuberculosis patients, Gaborone, Botswana, 2012–2016.
